# Changes to mineral levels in the yolk of meat chicken embryos during incubation

**DOI:** 10.3382/ps/pey423

**Published:** 2018-11-30

**Authors:** R L Hopcroft, A J Cowieson, W I Muir, P J Groves

**Affiliations:** 1 Poultry Research Foundation, Faculty of Science, The University of Sydney, Camden, NSW 2570, Australia; 2 DSM Nutritional Products, Wurmisweg, CH-4303 Kaiseraugst, Switzerland

**Keywords:** mineral, yolk, incubation, embryo, meat chicken breeders

## Abstract

A total of 864 settable Cobb 500 eggs were used to explore changes in yolk mineral content during incubation. Eggs were individually weighed and then placed in a commercial incubator. On embryonic day (ED) 0, 6.5, 13.5, and 17.5, 36 eggs were sampled and yolk weight and mineral content were determined. The concentration of iron (Fe), phosphorus (P), and zinc (Zn) declined (*P* < 0.05) from ED0 to ED17.5. The concentration of calcium (Ca), magnesium (Mg), and strontium (Sr) increased (*P* < 0.05) from ED0 to ED17.5. The concentration of copper (Cu), potassium (K), and sodium (Na) increased initially (ED0 to ED6.5) but declined thereafter. There was no change (*P* > 0.05) in the concentration of yolk manganese (Mn) from ED0 to ED17.5. Substantial changes in yolk mineral concentration occur during incubation and are presumably associated with mobilization of shell reserves and flux between albumen and yolk. These data may be useful in designing in ovo interventions, optimizing meat chicken breeder premix formulation or assembly of suitable neonatal or pre-starter diets for meat chicken chicks.

## INTRODUCTION

In poultry, embryonic fitness is reliant on adequate enrichment of the egg with the necessary amino acids, lipids, carbohydrates, and minerals from the parent hen to support optimal development (Romanoff, 1967; Moran, 2007). Transfer efficiency of these essential nutrients to the egg varies and this depends on the nutrition, fecundity, age, and health status of the breeders and the presentation of these nutrients in the diet e.g., organic compared with inorganic minerals (Angel, 2007; Yadgary et al., 2011). At lay the egg becomes an independent entity and there are limited options for intervention to fortify the developing embryo with additional macro- or micronutrients (Yair et al., 2015). Thus, it is of critical importance to ensure that the fertile egg contains a suitable repository of nutrients to maximize embryonic viability.

The isolated nutrient reserve of the fertile egg consists of 3 distinct nutrient compartments that are mobilized as required to support embryonic development, namely the shell, the yolk, and the albumen (Richards and Packard, 1996). These nutrient reservoirs operate dynamically during incubation and serve to support different stages of growth with provision of lipids, amino acids, carbohydrates, and minerals. In the case of minerals, the shell is the major source of Ca, Mg, and Sr; the albumen supplies the majority of K and Na; and the yolk provides Cu, Fe, Mn, P, and Zn (Richards, 1997; Schaafsma et al., 2000; Yair and Uni, 2011). Mobilization of mineral reserves from these distinct repositories occurs at different times during incubation. The extent of the success of this process will depend on qualitative and quantitative mineral status of the egg at lay. Furthermore, immediately post-hatch the chick relies on its residual yolk to provide nourishment until exogenous nutrient intake begins. Thus, an appreciation for the dynamics of mineral mobilization and utilization during incubation may give insight into suitable neonatal feeding strategies (to complement, rather than antagonize, residual yolk supply) as well as identify opportunities for intervention in ovo or in breeder nutrition. Yair and Uni (2011) identified low consumption of Cu, Mn, Zn, and Fe from the yolks of embryos prior to hatch, in eggs laid by 50-wk-old Cobb 500 meat chicken breeder birds. These authors concluded that modern meat chicken breeder hens, with adequate dietary mineral levels, may be unable to deposit adequate levels of these minerals into the egg. This has been suggested to lead to embryos which experience deficiencies in these minerals prior to hatching. In later work, bone ash in chickens at 38 d of age was increased following in ovo feeding of vitamin D_3_, Cu, K, Mn, Na, P, and Zn at ED17 (Yair et al., 2015).

It was therefore the purpose of the experiment reported herein to systematically assess the mineral composition of egg yolk from commercial meat chicken breeders throughout the incubation period. Experimental similarities allow a comparison to be drawn between this experiment and that of Yair and Uni (2011). This experiment measured strontium in the yolk during incubation for the first time. The presentation of yolk values for 10 minerals during incubation is novel and allows for greater comprehension of the movement of minerals into and out of the yolk during incubation.

## MATERIALS AND METHODS

All experimental procedures were approved by the University of Sydney Animal Ethics Committee (protocol number 2014/729) and strictly complied with the Australian Code of Practice for the Care and Use of Animals for Scientific Purposes as prepared by the Nation Health and Medical Research Council (2013).

### Incubation

A total of 864 settable Cobb 500 meat chicken eggs were obtained from a commercial hatchery (Cordina hatchery, Greystanes, NSW, Australia), laid by a breeder flock of 53 wk of age. Eggs that were cracked, dirty, or deformed were excluded from the study before incubation commenced.

Eggs were weighed individually and held at 17°C for 6 d. During this time a temperature sensor (Remote Intelligent Multisensors: TSIC 716 Advanced Sensor Technology; Netic Pty Limited, Ryde, NSW, Australia) was attached to a central egg of each tray. Contact of the sensor with the egg shell equator was maintained using thermal conductive paste (silicone heat transfer compound; Unick Chemical Corp., Gimhae, Republic of Korea). The sensors were connected to a remote physical monitor (Uptime Devices, Ryde, NSW, Australia) and to a notebook computer where a software program (Net Sensor Man^TM^; Netic Pty Limited) recorded temperatures continuously at 1-min intervals. The storage room temperature was increased to 25°C over 10 h prior to eggs being placed into 1 of 6 288-egg-capacity incubators (E2A—Multiquip Pty Limited, Austral, NSW, Australia) (144 eggs per incubator distributed on 4 trays of 36 eggs). Incubators were adjusted manually to maintain an egg shell temperature of 37.8°C according to the sensor readouts throughout the first 17.5 d of incubation. Eggs were consistently within 0.2°C of 37.8°C, with the exception of 2 incubators cooling to 37.5°C between embryonic day (**ED**) 8 and ED11, and 2 different incubators heating to 38.3°C on ED16. Relative humidity was maintained between 50 and 60% throughout by evaporation. Eggs were automatically turned 90° each hour.

### Sample Collection

At 0, 6.5, 13.5, and 17.5 ED, 6 eggs were sampled from each incubator (36 total) for fresh yolk weight and yolk mineral analysis. A small hole was cut around the wide end of the egg, where the air cell was located. The embryo was removed and euthanized. The yolk, including the yolk sac membrane and sub-germinal fraction, was poured out for collection.

### Mineral Analysis

To assess concentrations of Ca, Cu, Fe, K, Mg, Mn, Na, P, Sr, and Zn in yolk samples, the samples were initially homogenized, weighed, and freeze dried. Samples were then reweighed to give dried yolk weights. Powdered sample, weighing 0.5 g, was placed into a 50 mL digestion vessel, to which 5 mL of nitric acid was added. Samples were heated on a heat block at 50°C for 2 h, covered by a watch glass. Temperature was then raised to 90°C for 30 min. Samples were cooled to room temperature and 2 mL of hydrogen peroxide was added. Samples were then heated back to 90°C for 10 min, then heat was increased to 120°C for 30 min. Samples were finally diluted with distilled water to 30 mL. After preparation, samples were subjected to inductively coupled plasma optical emission spectroscopy (iCAP 6000 Series) according to manufacturer's instructions (Thermo Electron Corporation, Waltham, MA). From this, measured concentrations of these 10 minerals were obtained. Yolk mineral concentrations were multiplied by dried yolk weight to determine the absolute amount of mineral in each yolk. Dry yolk weight was subtracted from fresh yolk weight to give approximate water content.

### Statistical Analysis

Analysis was performed using the SAS Enterprise Guide 6.1 software package (SAS Institute Inc., Cary, NC). The data were analyzed by 1-way ANOVA for significance of differences and then the Tukey's HSD test was used to determine the differences between individual analytical terms (ED). Data from infertile eggs were not included in the analysis.

Mineral concentrations and absolute values were plotted against ED and a line of best fit added to the resulting graphs. The R^2^ value of these lines was recorded.

## RESULTS

Sampled egg physical characteristics are presented in Table 1. Fresh yolk weight and approximate water content increased (*P* < 0.05) from ED0 to ED6.5, and then decreased. Dry yolk weight significantly decreased at each sample point, except for between ED6.5 and ED13.5 where yolk weight did not appear to change significantly (*P* < 0.05).

**Table 1.** Sampled egg mean characteristics (±SE) through incubation.

Characteristic (g)	Embryonic day 0	Embryonic day 6.5	Embryonic day 13.5	Embryonic day 17.5
N=	35	29	26	18
Whole egg weight	65.25 (±0.83)	63.53 (±0.69)	62.41 (±0.80)	60.61 (±0.97)
Fresh yolk weight	23.03^b^ (±0.28)	27.2^a^ (±0.93)	18.62^c^ (±0.75)	15.87^d^ (±0.51)
Dry yolk weight	11.01^a^ (±0.14)	8.33^b^ (±0.31)	8.09^b,c^ (±0.35)	7.27^c^ (±0.26)
Yolk water content	11.86^b^ (±0.17)	18.72^a^ (±0.71)	10.86^b^ (±0.51)	8.43^c^ (±0.28)

^a-d^Means within a row with different superscript significantly differ (*P* < 0.05).

Eggs laid by a 53-wk-old Cobb 500 parent breeder flock.

The concentrations of minerals in the yolk are presented in Table 2. Yolk Ca and Sr concentrations increased (*P* < 0.001) from ED6.5 to ED13.5, and again at ED17.5. Yolk Cu, K, and Na concentrations increased (*P* < 0.001) from ED0 to ED6.5, then decreased to ED13.5. Yolk Fe and P concentrations decreased (*P* < 0.001) from ED6.5 to ED13.5, and again at ED17.5. Yolk Mg concentration significantly increased (*P* < 0.001) at each sample time. Yolk Mn concentration did not change during incubation. Yolk Zn concentration decreased (*P* < 0.001) from ED13.5 to ED17.5.

**Table 2.** Mean yolk mineral concentrations (±SE) through incubation.

Mineral (mg/kg)	Embryonic day 0	Embryonic day 6.5	Embryonic day 13.5	Embryonic day 17.5
N=	35	29	26	18
Ca	2676^c^ (±30)	2799^c^ (±111)	4714^b^ (±395)	7033^a^ (±409)
Cu	2.77^b^ (±0.04)	3.03^a^ (±0.06)	2.51^c^ (±0.08)	1.35^d^ (±0.06)
Fe	116^a^ (±2.09)	108^a^ (±4.33)	67^b^ (±2.71)	38^c^ (±2.31)
K	1771^c^ (±22)	3074^a^ (±93)	2526^b^ (±93)	2349^b^ (±78)
Mg	234^d^ (±2.38)	269^c^ (±6.28)	311^b^ (±7.11)	342^a^ (±6.82)
Mn	2.52 (±0.11)	2.55 (±0.12)	2.06 (±0.13)	2.09 (±0.21)
Na	954^d^ (±12)	4103^a^ (±189)	2199^b^ (±81)	1419^c^ (±90)
P	10426^a^ (±40)	10872^a^ (±182)	9478^b^ (±176)	7606^c^ (±138)
Sr	2.61^c^ (±0.06)	2.62^c^ (±0.13)	3.56^b^ (±0.31)	4.57^a^ (±0.33)
Zn	67.1^a^ (±0.68)	69.6^a^ (±2.85)	66.8^a^ (±3.01)	47^b^ (±1.44)

^a–d^Means within a row with different superscript significantly differ (*P* < 0.001)

Yolk collected from eggs laid by a 53-wk-old Cobb 500 parent breeder flock.

Absolute yolk mineral value contents are shown in Table 3. Absolute yolk Ca increased (*P* < 0.05) from ED6.5 to ED13.5, and again at ED17.5. Absolute yolk Cu, Fe and P decreased (*P* < 0.05) at each consecutive sample time. Absolute yolk K and Na increased (*P* < 0.05) from ED0 to ED6.5, then decreased. Absolute yolk Mg decreased (*P* < 0.05) from ED0 to ED6.5, then numerically increased at ED13.5. Absolute yolk Mn decreased (*P* < 0.05) from ED0 to ED6.5, and again at ED17.5. Absolute yolk Sr decreased (*P* < 0.05) from ED0 to ED6.5, then increased at ED13.5. Yolk Zn decreased (*P* < 0.05) from ED0 to ED6.5, and again from ED13.5 to ED17.5.

**Table 3.** Mean yolk absolute weight (±SE) through incubation.

Mineral	Embryonic day 0	Embryonic day 6.5	Embryonic day 13.5	Embryonic day 17.5
Ca (mg)	29^c^ (±0.6)	23^c^ (±1.3)	37^b^ (±3.4)	50^a^ (±2.6)
Cu (μg)	30.55^a^ (±0.62)	25.09^b^ (±0.99)	19.94^c^ (±0.81)	9.94^d^ (±0.71)
Fe (mg)	1.28^a^ (±0.028)	0.91^b^ (±0.054)	0.53^c^ (±0.028)	0.28^d^ (±0.022)
K (mg)	19.5^b^ (±0.34)	25.4^a^ (±1.06)	20.2^b^ (±1.06)	17^b^ (±0.74)
Mg (mg)	2.58^a^ (±0.049)	2.24^b^ (±0.095)	2.5 (±0.109)	2.48 (±0.104)
Mn (μg)	27.85^a^ (±1.34)	21.37^b^ (±1.36)	16.75^b,c^ (±1.37)	15.33^c^ (±1.8)
Na (mg)	10.5^c^ (±0.18)	33.7^a^ (±1.75)	17.6^b^ (±0.87)	0.4^c^ (±0.8)
P (mg)	115^a^ (±1.5)	91^b^ (±3.9)	76^c^ (±3.2)	55^d^ (±2.4)
Sr (μg)	28.68^a^ (±0.71)	22.06^b^ (±1.42)	28.79^a^ (±3)	32.54^a^ (±2.08)
Zn (mg)	0.74^a^ (±0.013)	0.58^b^ (±0.031)	0.52^b^ (±0.017)	0.34^c^ (±0.018)

^a–d^Means within a row with different superscript significantly differ (*P* < 0.05).

From ED0 to ED17.5, absolute yolk Ca increased by 71%, Sr increased by 13%, Na decreased by 1%, Mg decreased by 4%, K decreased by 13%, Mn decreased by 45%, P decreased by 52%, Zn decreased by 54%, Cu decreased by 67%, and Fe decreased by 78%.

**Figure 1.**
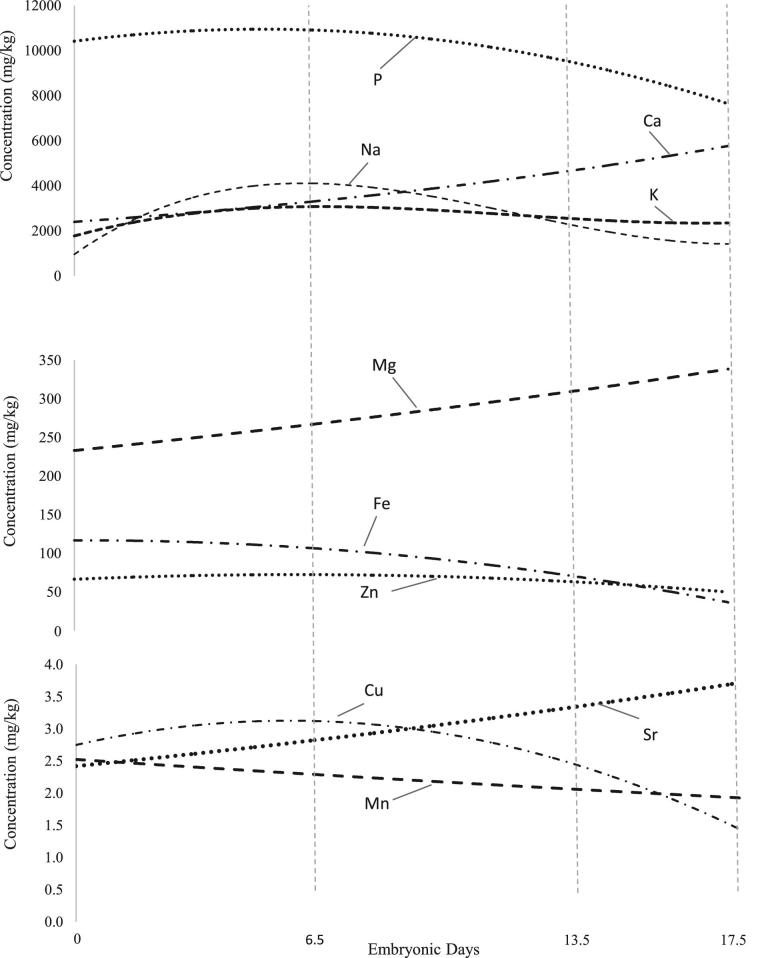
Regression lines of yolk mineral concentrations against time. Regression lines for P, Fe, Zn, and Cu are binomial. Regression lines for Ca, Mg, Mn, and Sr are exponential. Regression lines for Na and K are trinomial.

Regression lines for mineral concentrations against embryonic day are shown in Figure 1. Concentrations of Ca and Mg increase exponentially over time (*P* < 0.001, R^2^ = 0.65 and 0.68, respectively). The concentration of Sr increases binomially over time (*P* < 0.001, R^2^ = 0.5). Concentrations of Cu, Fe, P, and Zn decrease binomially over time (*P* < 0.001), with stronger relationships for Cu, Fe, and P (R^2^ > 0.7). The concentration of Mn decreases exponentially over time (*P* < 0.001, R^2^ = 0.1). Concentrations of K and Na change trinomially over time (*P* < 0.001, R^2^ = 0.83, 0.65, respectively).

Absolute Ca, Mg, and Sr change binomially over time (*P* < 0.001, R^2^ = 0.43, 0.05, 0.22, respectively). Absolute Cu, Fe, P, and Zn decrease linearly over time (*P* < 0.001, R^2^ > 0.57). Absolute Mn decreased exponentially over time (*P* < 0.001, R^2^ = 0.31). Absolute K and Na change trinomially over time (*P* < 0.001, R^2^ = 0.32, 0.76, respectively).

## DISCUSSION

Yolk Ca, Cu, and P content at ED0 was similar to the values reported by Yair and Uni (2011). However, in the current trial, yolks had slightly higher Mn, 50% lower Fe, and 75% lower Zn compared to those reported by Yair and Uni (2011). Those authors reported low yolk Cu, Mn, Zn, and Fe consumption by embryos prior to hatch.

Yolk mineral content at ED0 can be modified by changing mineral levels in the breeder diet, with little effect for some minerals (Richards, 1997; Angel, 2007). Providing the mineral in an organic form compared to an inorganic form can influence progeny performance (Kidd, 2003; Torres, 2013). For example, feeding organic Zn-methionine to breeders rather than inorganic ZnSO_4_ produced chicks with better cellular immunity and heavier bone weight (Kidd et al., 1992). However, hens fed a Zn-amino acid complex laid eggs which had higher early embryonic mortality compared to hens fed ZnSO_4_ (Hudson et al., 2004). In that experiment, feeding a mixture of the Zn-amino acid complex and ZnSO_4_ resulted in the highest hatchability.

At ED17.5, yolk from this experiment contained approximately 124, 144, 155, 300, 340, and 470% more Fe, Ca, P, Cu, Mn, and Zn, respectively, than that recorded at ED17 by Yair and Uni (2011). This discrepancy could be due to differences in breeder hen nutrition.

The 10 minerals observed in this experiment can be divided into 3 groups based on initial egg fraction location, patterns in changes to absolute yolk mineral levels during incubation, and regression analysis. Cu, Fe, P, and Zn form one group: they are predominantly found in the yolk, the yolk concentration of these 4 minerals decreases binomially over time, and the absolute value of each decreases in a strong linear fashion over time.

Movement of minerals from the yolk is facilitated via endocytosis by the yolk sac membrane (Mobbs and McMillan, 1981; Bauer et al., 2013). Analysis of the data has revealed changes in mineral absorption rates during different periods of incubation. These changes can be linked to other research in the area, improving understanding of the mechanisms at work in yolk absorption. For example, absolute yolk Zn did not significantly decrease between ED6.5 and ED 13.5; a yolk sac membrane Zn transporter (ZnT-1) has been shown to decrease in relative expression during the same period (Yadgary et al., 2011). The only observed decrease in Zn concentration occurred from ED13.5 to ED17.5; expression of ZnT-1 increases in the yolk sac membrane during this period (Yadgary et al., 2011).

K and Na form a second group. Both minerals are initially found predominately in the albumen (Simkiss, 1980), and change in K and Na concentrations and absolute values are best described trinomially.

The absolute values and concentrations of yolk K and Na increased during the first week of incubation. The albumen is high in water, K, and Na (Yair and Uni, 2011); the blastoderm absorbs water, K, and Na from the albumen via its ectodermal surface and secretes them through the endodermal surface into the yolk (New, 1956; Simkiss, 1980). This period of albumen influx ensures adequate yolk Na, K, and water reserves for embryonic utilization during incubation and after hatch. This period of albumen influx could potentially provide an advantage in planning in ovo injections at ED0 if eggs are known to be lacking in minerals or other nutrients.

Calcium, Mg, and Sr comprise the third group: they each decreased in absolute amount from ED0 to ED6.5, and increased from ED6.5 to ED13.5. They also increased in concentration over time. Within this group, Ca and Sr seem more strongly associated to each other than Mg. Changes to the absolute values of these minerals over time can be described using binomial equations.

Increases in yolk Ca and Mg during incubation have been shown to be facilitated by the chorioallantoic membrane transporting minerals from the eggshell (Johnston and Comar, 1955; Cheville and Coignoul, 1984; Gabrielli and Accili, 2010; Uni et al., 2012). As the shell contains Sr (Schaafsma et al., 2000), the increase of yolk Sr during this same period may be due to mobilization of shell Sr by the chorioallantoic membrane, and its subsequent deposition in the yolk.

Eggshell Sr can be increased via Sr supplementation to the laying hen (Browning and Cowieson, 2015). Increasing dietary Sr to 1,000 mg/kg decreased eggshell Mg, whereas a level of 500 mg/kg had no significant effect on eggshell Mg (Browning and Cowieson, 2015). Strontium supplementation in meat chickens has the potential to improve the performance, mineral retention, and bone characteristics of meat chickens (Browning and Cowieson, 2014). Increased dietary Mg increased eggshell Mg in layers (Ding and Shen, 1992). Increasing dietary Sr thus could increase yolk Sr levels during the later stages of incubation via higher availability in the shell. The effects of balanced additional Mg and Sr in the diet of meat chicken breeder hens could have a positive effect on progeny performance and bone mineralization, and should be the subject of further investigation. However, these need to be carefully managed as adding dietary Mg can affect Ca and P absorption (Shastak and Rodehutscord, 2015).

Manganese is predominately found in the yolk; however, it is also present in the shell (Yair and Uni, 2011). Manganese was the only mineral which did not significantly change in yolk concentration throughout incubation; however, absolute yolk Mn decreased exponentially over time. Relatively little is known about Mn storage in yolk or transport of Mn through the yolk sac membrane (Grau et al., 1979; Richards, 1991), and research designed to understand the usage and storage of embryonic yolk Mn may reveal unexpected mechanisms at work.

Interestingly, the grouping of the minerals outlined above corresponds to the location of elements on the periodic table. Potassium and Na, undertaking albumen efflux, are both in the alkali group; Mg, Ca, and Sr, which are mobilized from the shell, are in the alkaline earth metals group; Cu, Fe, Mn, and Zn are group 4 transitional metals, and P is a non-metal. This observation may be relevant to ion transportation across the yolk sac membrane.

This experiment provides a description of changes in yolk mineral concentration and absolute quantities during incubation. This information may serve as a benchmark for studies which focus on both incubation, and the immediate time period after hatch, when chicks depend on their residual yolk sac as a nutritional source. The movement of Sr from the eggshell needs to be confirmed, and possible benefits of Sr supplementation in breeder diets explored. Given the plasticity of mineral levels during incubation, opportunities to manage these levels to more closely meet the needs of the embryo, for example the effects of changed incubation conditions, breeder diet mineral inclusion levels, and the age of breeder flock, are worthy of further investigation. The baseline values and ranges established in this study will allow for future work to be set in appropriate context.
